# Learning from implementation of a COVID case management desk guide and training: a pilot study in Sierra Leone

**DOI:** 10.1186/s12913-023-10024-6

**Published:** 2023-09-25

**Authors:** Sophie Witter, Guanyang Zou, Kiran Cheedella, John Walley, Haja Wurie

**Affiliations:** 1 NIHR Research Unit on Health in Situations of Fragility, Institute for Global Health and Development, Queen Margaret University, Edinburgh, UK; 2 School of Public Health and Management, Guangzhou University of Chinese Medicine, Guangzhou, China; 3 Nuffield Centre for International Health and Development, Leeds Institute for Health Sciences, University of Leeds, Leeds, UK; 4 College of Medicine and Allied Health Sciences, University of Sierra Leone, Freetown, Sierra Leone

**Keywords:** COVID-19, Sierra Leone, Case management, Diagnosis, Infection prevention and control, Essential services, Primary care

## Abstract

**Background:**

When the COVID pandemic hit the world, there was need for applied guides and training materials to support frontline health care staff to manage patients effectively and safely and to educate themselves and communities. This article reports on the development and piloting of such a set of materials in Sierra Leone, which were based on international evidence but adapted to the local context. Reflecting on this experience, including community and health system barriers and enablers, is important to prepare for future regional shocks.

**Methods:**

This study, in Bombali district in 2020, piloted user-friendly COVID guides for frontline health workers (the intervention), which was evaluated using facility checklists (pre and post training), routine data analysis and 32 key informant interviews.

**Results:**

Key informants at district, hospital and community health centre levels identified gains from the training and desk guides, including improved diagnosis, triaging, infection prevention and management of patients. They also reported greater confidence to share messages on protection with colleagues and community members, which was needed to encourage continued use of essential services during the pandemic. However, important barriers were also revealed, including the lack of testing facilities, which reduced the sense of urgency, as few cases were identified. Actions based on the Ebola experience, such as setting up testing and isolation centres, which the community avoided, were not appropriate to COVID. Stigma and fear were important factors, although these were reduced with outreach activities. Supplies of essential medicines and personal protective equipment were also lacking.

**Conclusion:**

This pilot study demonstrated the relevance and importance of guides adapted to the context, which were able to improve the confidence of health staff to manage their own and the community’s fears in the face of a new pandemic and improve their skills. Previous epidemics, particularly Ebola, complicated this by both creating structures that could be revitalised but also assumptions and behaviours that were not adapted to the new disease. Our study documents positive adaptations and resilience by health staff but also chronic system weaknesses (particularly for medicines, supplies and equipment) which must be urgently addressed before the next shock arrives.

**Supplementary Information:**

The online version contains supplementary material available at 10.1186/s12913-023-10024-6.

## Background

The COVID pandemic is increasingly threatening global health, economic development and social stability, especially in fragile countries. The World Health Organisation (WHO) Africa Region has reported nearly 9 million confirmed cases, including more than 174,000 deaths, as of August 2022 [1]. As of 11th August 2022, Sierra Leone has reported nearly 7,738 cases of COVID [2]. However, a nationally representative survey in Sierra Leone showed seroprevalence was 2.6%, and an estimated 203,060 infections by March 2021 [3], at least 26 times higher than the reported number of cases. Although many lessons were learned from the country’s fight against Ebola in 2013-14, Sierra Leone, as a post-conflict and fragile state, has encountered many institutional and operational challenges in its response to the pandemic. Challenges included the inadequate resources and processes for contact tracing to enable surveillance and control of virus spread. Further, by redirecting limited resources to the pandemic response, the Government of Sierra Leone risked compromising the ability of the existing health system to offer routine health services [4].

Literature on COVID in Africa has mainly focused on prediction and modelling of the COVID epidemiology [5–11]. Many studies have analysed the capacity of health systems in Africa to respond to the pandemic, highlighting some positive experiences and strengths, such as technical leadership and multi-sectoral responses [12], positive effect of non-pharmaceutical interventions [13, 14], implementation of Infection Prevention and Control (IPC) Committees [15], primary care approaches and benefits of technological innovations [16]. However, many studies also highlighted limited capacity to control the COVID pandemic [17], including poor testing capacity, under-funded health systems, lack of Intensive Care Unit beds, shortages of ventilators, personal protective equipment (PPE) for health workers, medical equipment, and medicines. Efforts on outbreak responses were further undermined in conflict-affected countries due to damaged infrastructure, depleted workforces, and limited funding [17]. Hagan et al. emphasized the need for public health infrastructure development (e.g., laboratories, infectious disease centres, regional hospitals), human capacity building, and regular public health educational campaigns for combating COVID and potential future outbreaks [6]. Other barriers to response efforts include lack of availability of water sanitation and hygiene (WASH) services in health facilities [18], challenges to re-organisation of care delivery [19] and practical challenges such as social distancing and quarantine in the economic and social context of Africa [20]. While lockdown measures designed to control the prevalence, interrupted health services access and caused social and economic disruption [21]. Schmidt et al. also document that false information circulated on social media not only caused confusion, fear and panic, but also contributed to the construction of misconceptions and stigmatizing responses to COVID [22]. The fragile health system and developmental differences, require a tailored approach to COVID-19 case management in African countries [23].

To support the health system’s responses to COVID in Sierra Leone, the research team developed systematic prevention and control management desk-top guides, training materials and records on COVID. These were adapted for use by health workers based in hospitals, Community Health Centres (CHCs) and other Peripheral Public Health Units (PHUs) in Sierra Leone. The desk guides were designed to improve systematic prevention and control (identification of possible COVID, diagnose, treat and educate) and health facility coordination to respond to COVID effectively at district hospital and CHC level. This article reports on an assessment of their feasibility in order to guide future adaptation and nationwide roll-out in this and similar contexts. The paper aims to fill a gap in studies addressing the issue of protocols, guides and training for management of COVID and similar epidemics, particularly at primary health care (PHC) level in low- and middle-income (and fragile) settings.

## Methods

This study was conducted in Bombali district, where the research team has worked in the past four years, including pilot-testing NCD desk-guides [24]. It was based on an action research project, covering the district hospital and CHCs, to pilot and roll out desk-guides and track any issues linked to their implementation. The implementation was embedded within routine health service delivery systems. Training and introduction of desk-guides started from June to December 2020. The Ministry of Health and Sanitation (MoHS), with support from the research team, was to consider scaling up of the desk-guides and training according to the results of the feasibility assessment.

In this section, we first describe the development of the intervention and then the study data collection methods, which were mixed, including qualitative (key informant interviews) and quantitative (routine data analysis) in Bombali district over 2020–2021, analysed thematically for the qualitative data and using Excel for the quantitative and then combined to answer the research questions.

### The intervention

The intervention was designed to fill a gap in user-friendly guides and training for frontline health workers. The MoHS in Sierra Leone usually adapts guidelines from WHO – e.g., for IPC, inpatient care, isolation centres. However, there were no guides and tools for the front-line outpatient care settings, where people attend with common symptoms, that might be COVID (e.g. fever, cough, breathlessness). There was a need for guides, training modules and record cards adapted for front-line outpatient health workers.

All materials were based on COVID guidelines related content from WHO, Sierra Leone, China and the UK^1^, adapted for the primary care facilities in LMICs. The guide covered case identification, diagnosis, management, referral, education for home isolated cases, communication with the district and community health worker, self-protection and facility management. The guide recognises that some COVID symptoms such as fever and cough are common to other febrile/ respiratory illnesses. Additionally, it draws attention to those at higher risk such as persons suffering from non-communicable diseases (NCDs) such as hypertension and diabetes. It also supports continuum of care for all patients, including COVID patients.

The developed guides and training modules were reviewed by clinical specialists at national level and by the District Health Management Team (DHMT), led by the District Medical Officer (DMO). Printed PHC guides [25] were disseminated and later made available electronically and online via the MoHS website. Social media platforms (e.g., WhatsApp) were also used by the health workers to ensure that the reach of the developed resources was wide.

A training plan for the Community Health Officers (CHOs) was co-created by the research team and the DHMT. One lead CHO trained others on the guide and training modules in a one-day training in August 2020, including 22 health workers (21 CHOs and one midwife; 15 males and 7 females). Myths concerning COVID, facts on its diagnosis, management and prevention, as well as how to use the limited available resources in the health centres to curtail the disease, were all discussed. Role playing and group discussions ensured a participatory, interactive approach. Training included case studies and role plays with various clinical and social example cases. The cases included how to identify likely COVID from other febrile and respiratory conditions, access COVID tests where available, and educate on home isolation and prevention of transmission and to identify and escalate care for patients with severe disease. This skill-based small group case study exercise approach has been tried and tested, with guides for other groups of diseases, for clinical decision and communication skills. The guide included how to triage and separate the flow of patients attending with symptoms that could be COVID from others; on IPC - to protect patients and health workers, whilst ensuring continuum of other routine services (e.g. immunisation and antenatal care (ANC)).

The CHOs also created a WhatsApp group, including members of the research team, in which questions could be posed to the wider group, and clarifications and solutions to problems posted. The lead CHO was primarily responsible for offering answers and clarifications to group questions and the research team took responsibility for collating relevant information from the group and adapting guides as necessary to ensure fit to context. Interviews with key informants (see below) also informed the adaptions.

The hospital guide covered screening and isolation area management, clinical treatment, healthcare staff management, hospital work plan requirements, IPC for COVID, and management of hospital supplies. This guide was developed to help hospital managers plan the layout of their hospital to cope with COVID, and continue management of other important health conditions and implement prevention programs. It complemented the Sierra Leone case management standard operating procedures. After review by the Medical Superintendent it was taken up by Bombali district hospital.

### Data collection

The study used mixed methods, including key informant interviews to collect data on district, organisational and clinical challenges and responses, and the effectiveness of the piloted tools in addressing these. We additionally used structured checklist assessments at facility level and triangulated findings of these two against routine data collected within the health system. Data collection took place between July 2020 and February 2021.

#### Key informant interviews (KII)

The KIIs aimed to understand: 1) the changing context (e.g. the epidemic’s progress and changes in government directives and resources for it); 2) how the guides had been used (including barriers and enablers of implementation); 3) what action they prompted in relation to COVID control, service delivery and its acceptability; and 4) the role of guides in relation to routine service delivery, e.g. considering continuity of NCD and ANC care. Lessons learned from interviews were fed into adaptation of guides and recommendations for further scaling.

Sampling for participants was purposive, based on participants’ expertise and involvement in COVID response and routine service delivery. Provided professionals had been in their role for more than 6 months, they were eligible to take part in interviews. Sampling was convenience based in that we were flexible in relation to potential participants’ time availability: we sought not to disrupt care delivery and scheduled interviews at a convenient time for participants.

Data collection was conducted in phases, with a small set of informants being repeatedly interviewed to track changes over the period of introduction of the guides (see Supplementary file 1 for interview topic guides). In the first and last round of interviews, we interviewed 19 and 9 KIIs respectively. In the interim, we interviewed a small pool of key informants(*n* = 4) from district, hospital and CHCs, who were especially well informed and willing to talk regularly. In total, we conducted 32 such interviews.

#### Quantitative data collection

We additionally embedded data collection in routine processes, and used routine health system data as much as possible to inform the research. First, CHO mentors visited 21 CHCs that participated in the training. During these visits, mentors were asked to complete a checklist which aimed to assess changes made using the PHC COVID desk guide (see Supplementary file 2). Secondly, we collected data from the national health management and information system (HMIS) to analyse trends in wider service use and how that had been impacted by COVID.

### Data analysis

#### Qualitative data analysis

All interviews were audio-recorded with consent of the interviewees, and transcribed verbatim. The framework method was used for analysis. Analysis focused on the research aims listed for KIIs and sought to understand the feasibility of implementing the care as per the desk-guides in the hospital and CHCs for COVID control and to maintain routine services, and the contextual factors which influenced these results.

#### Quantitative data analysis

Descriptive statistics were used to analyse the national HMIS data to understand trends in wider service use in the district and nationally, comparing the 2019 with 2020 data. For the facilities checklist, we compared data from June 2020 with September 2020 (before and after the training was conducted).

## Results

The results start by examining the perceived benefits of the COVID guides at CHC and hospital levels in relation to COVID itself but also maintenance of routine services. This is followed by an examination of the barriers and facilitators to their uptake, including health system factors. The findings are summarised in Table 1.

**Table 1:** Summary of findings

Themes and subthemes			Key messages
**Perceived benefits** **of guides**	**Management of COVID cases**		• Improved diagnosis skills and IPC(CHC) • Improved triaging of patients (CHC) • Improved diagnosis, IPC and treatment, better communication with patients (hospital) • Greater confidence in managing patients (hospital) • Increased awareness of the need for isolation of suspected cases (hospital)
	**Staff protection**		• Improving providers’ knowledge, awareness in managing COVID cases and protecting themselves (CHC/hospital)
	**Routine services**		• A drop and then a recovery in many of the routine clinical services (hospital) • Encouraging the continuation of the routine health services (district)
	**Patient awareness**		• Improving patient awareness of protection and social distancing, as well as reduced stigma for COVID (CHC) • Compliance with mask-wearing was reported as declining (hospital)
**Facilitators and barriers**	**Barriers**	**For use of guides**	• Lack of screening, testing and identification of COVID cases (CHC) • Limited space and equipment (CHC) • Concern about the difficulty of diagnosing COVID (CHC) • Lack of guidance on addressing the concerns of patients and diagnosis and treatment guides for other conditions.
		**Community concerns**	• Patients were afraid of coming to the clinic as they are afraid of being infected by health staff and of being diagnosed with COVID and referred to the Government Regional hospital or isolated.(CHC) • Isolation is especially problematic (CHC) • TB patients who had similar symptoms to COVID might avoid visiting the facility (CHC)
		**Drug supplies and equipment**	• Common essential drugs were described as out of stock (CHC) • Routine vaccines were perceived as being in short supply CHC) • Broader equipment and infrastructure challenges remained evident too (CHC)
		**Supply of PPE**	• There was very poor availability of PPE and health protection among health workers and patients (CHC and hospital)
		**Health workforce challenges**	• Lack of staffing, staff not on payroll and high staff turnover (CHC) • Lack of training on COVID prior to this intervention (CHC)
	**Facilitators**	**For adoption of guides**	• Action taken by trainees to share resources and knowledge (CHC) • Ongoing training and encouragement to use the desk-guide (CHC) • Actions also described to cascade knowledge to all groups of staff (hospital)
		**For management of COVID**	• Lots of community meetings were held to inform them about COVID and dispel fears (CHC) • Active collaboration between the DHMT and the district COVID coordination body (hospital) • Partner investment supported refurbishing the treatment centre and testing for COVID(hospital) • A substantial focus on IPC measures (hospital) • Sick people could break the law enforced curfew and attend the hospital for care (hospital)
		**For maintenance of routine services**	• Outreach campaigns improved the immunisation, ANC and under five care awareness and uptake (CHC) • Routine clinical services continued to be offered as before, including referrals as required (CHC) • Services were reorganised in response to COVID (CHC) • Sensitization was reported to help improve awareness and service use by residents (hospital).

### Perceived benefits of guides

The interviewees commented on the relevance of the desk guide care processes, which were seen as comprehensive and the first used to train CHOs on COVID in the district.

#### Management of COVID cases

CHOs highlighted improved diagnosis skills and IPC:

*“It is very relevant because there are some signs that are indicated in the desk[guide] which before this time we never knew about them, but through the desk guide we were told that whenever someone enter in the facility with a history of cough or contact with someone positive, or if the person is complaining loss of taste or smell with high fever, we can take that individual as a suspected case. So, through the desk guide we were able to know that” (CHC KI)*

The desk-guide also helped in some CHCs to improve the triaging of patients, separating patients with COVID related symptoms from others.

*“At first, we don’t have a triage but since we have done the training, I instituted a triage. And I have ensured that hand washing facilities are present in all units and before entering the facility.” (CHC KI)*

At the hospital level, improved diagnosis, IPC and treatment, better communication with patients, were highlighted by KIs.

*“Because it guides you how to go about your procedure, it helps you know how to disinfect, it also guides us about the five movement of hand washing, disinfection and all the skills. It also helps the staff to go about their work in a protective manner.” (Hospital KI)*

Staff highlighted that they were encouraged to be friendly and confident in managing patients, without neglecting safety measures.

*“Yes, because at first, we were afraid when they said COVID, because we were not knowledgeable, we just think when they come here at once we should refer them. Even not to come closer to them so it causes stigma to them. With the desk guide we learnt that we should be counselling them and be friendly to them but we should have two meters apart. We should be in our face mask and we should give them what they need before any result” (Hospital KI)*

Hospital staff also reported increased awareness of the need for isolation of suspected cases.

*“From what I learn from the desk guide, it tells us that you can isolate somebody in his/her own house, excluding his children and wife, monitoring them by phone, you can communicate, if there is no improvement you can call on the line for him or her to be picked up.” (Hospital KI)*

#### Staff protection

The desk guide was also reported to help improve the providers’ knowledge, awareness and confidence in managing COVID cases and protecting themselves, as reported at primary and secondary levels.

*“Yeah, before this time without the guide we don’t even know the type of patient we are dealing with, but since we have this guide now, we now know the questions we are asking to those patients… even the risks among us the staff have reduced.” (CHC KI)*

*“(Before) everybody was afraid, when they say COVID. Every staff was afraid… But now they all have confidence.” (Hospital KI)*

#### Routine services

The desk guide also encouraged the continuation of the routine health services.

*“We know that even with COVID, we can still give immunisation to beneficiaries of ANC. You can attend to the person with your PPE at a two-meter distance. Putting on your facemask you can communicate with the person. You can counsel the person, you can talk to relatives, you can reassure the person.” (Hospital KI)*

Key informants reported a drop and then a recovery in many of the routine clinical services.

*“Well, around last year up to March, April there was a significant reduction up to about 30 to 40% in clinical attendance and most of our interventions, there were drop in our coverages, about 25 to 30% drop in coverages on most of the intervention” (District KI)*

**Fig. 1:**
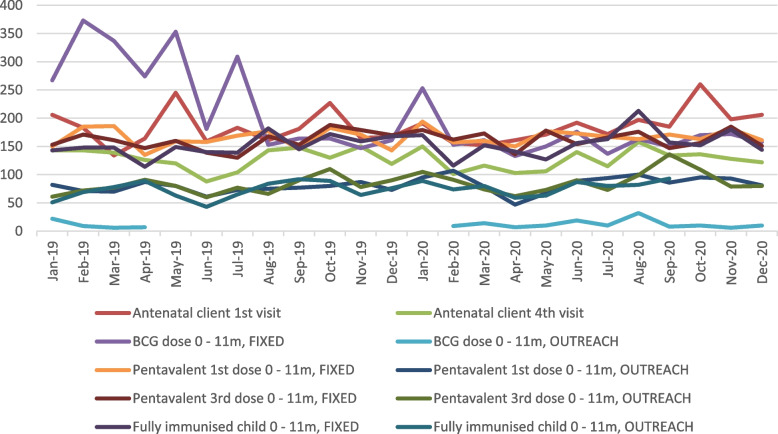
Trends in ANC and immunisation uptake, Bombali district, 2019–2020 *Source: DHMIS data*

**Fig. 2:**
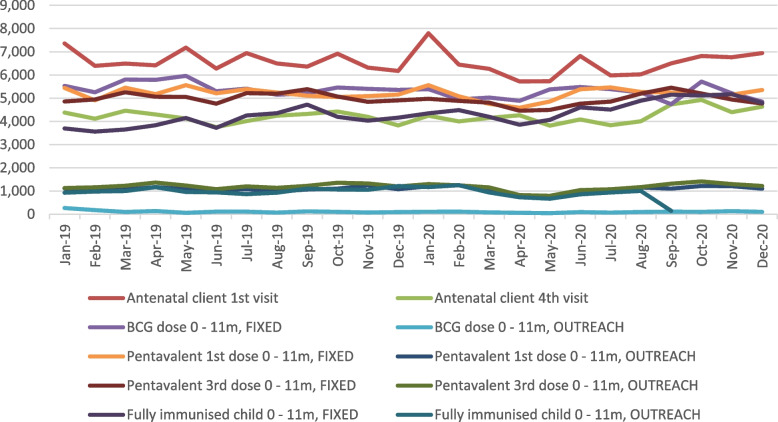
Trends in ANC and immunisation uptake, Sierra Leone, 2019–2020 *Source: DHMIS data*

HMIS data (Figs. 1 and 2) indicate that ANC and immunization visits at CHC level in Bombali district broadly held up during the COVID period (except for BCG), which is consistent with the wider national pattern (assuming that reporting is complete).

#### Patient awareness

Our participants also suggested that the intervention improved patient awareness of protection and social distancing, as well as reduced stigma for COVD.

*“The use of face masks has gone well. Since last month, we are not talking that much again about the use of face mask. Now, when patients are coming, they are holding them on their hands and they are wearing them as well. That has helped us seriously. Also, in the area of hand washing. Now even if they don’t tell them to wash their hands, the moment they see the hand washing bucket they will go there and wash their hands” (CHC* KI)

However, after eight months with low levels of testing and few confirmed cases in the district, compliance with mask-wearing was reported as declining.

*“Although most have changed, they don’t think it a major threat now, some have relaxed. They are not complying.” (Hospital KI)*

## Barriers and facilitators

### Barriers

#### For use of guides

All CHC informants highlighted the lack of screening, testing and identification of COVID cases as a challenge, leading to a lack of sense of urgency about COVID by providers.

*“I think the changes that I expect should have happen is to increase the testing and make more testing sites in Bombali district and at chiefdom level but since the start of COVID we still do not have chiefdom testing site.” (CHC KI)*

According to our tracking of the 21 CHCs that took part in the training, there were no confirmed COVID cases, presumably because of the lack of testing. Of the 7,588 patient visits recorded in the month of June 2020, 37% presented with fever and 23% with coughs, but only three cases reported loss of sense of smell or taste.

At Makeni (the district headquarter town) hospital, 31 COVID cases were recorded in the first six months of the pandemic, which remained low.

Limited space and equipment was one of the main practical barriers to implementing the provisions of the desk guide.

*“If you look at the facility you will see that the facility is small and it is not spacious. So ensuring one-meter distance could cause another patient to stand. More especially during Mondays and Fridays.” (CHC KI)*

It was discovered during the follow up visit to the 21 CHCs that 8 of the visited facilities had designated a triaging area. This was an increase from the one triage area reported before the training. In those triaging areas, there were tables and chairs but only half of them had thermometers and screening books, that were not being utilised at the time of the visit, as either there was no trigger or the person assigned was absent.

In relation to isolation, a few facilities were observed to be screening patients and putting patients with cough and high fever in separate places. All bar two CHCs had isolation units. However, more than 40% of those 19 isolation areas had functional issues, such as damaged windows, doors, lack of beds and leaking roofs. Isolation units had not been used in any case due to lack of confirmed cases. Some dated from the Ebola period but had since been used as storerooms or delivery rooms.

*“In terms of isolation, that it is not functional because it has no beds… you move the mattress to the isolation centre maybe, after the patient had recovered you wash it and bring it back.” (CHC KI)*

For IPC, all 21 visited facilities had hand-washing stations (an increase on the 13 that had them prior to the intervention) but only a few facilities reported to have surgical masks and hand sanitizers. Nine facilities reported to have a few gloves and all others had no gloves at the time of the visit (except gynaecological gloves that are used in the labour room).

Participants also expressed concern about the difficulty of diagnosing COVID, given the overlap of symptoms with other conditions and the absence of COVID tests.

*“If possible, we should just ensure that all suspected cases are transferred to treatment centres or they are transferred for testing; that will only be possible if we have rapid kits.” (CHC KI)*

More guidance on addressing the concerns of patients was suggested for the content of the guide, along with the need for more community sensitisation on COVID in general, and more specifically to address stigma. Staff also suggested that the guide should include diagnosis and treatment guides for other conditions.

#### Community concerns

There was a general perception that patients were afraid of coming to the clinic as they are afraid of being infected by health staff and of being diagnosed with COVID and referred to the Government Regional hospital or isolated. Suspicions and bad memories remained from the Ebola period and rumours played an important role in preventing patients and lactating mothers from visiting the CHCs.

*“People were saying that if they come to the CHC they will inject them with coronavirus. So they were not coming because of that. Except now, based on the sensitisation that is being provided, the turn out now is very huge.” (CHC KI)*

Isolation is especially problematic from a community perspective and also economically, given the need to continue to work. Separating patients with coughs was reported to be possible in some facilities, though with challenges as many of the patients felt discriminated against and felt that they were being marginalised by the CHOs when asked to sit in separate areas.

*“Option for self-isolation is not feasible in remotes areas as many patients will not sit at home for some illnesses, not to talk about fever and cough.” (CHC KI)*

Concern existed that TB patients who had similar symptoms to COVID might avoid visiting the facility for fear of being diagnosed as COVID patients.

*“In terms of TB the changes that where done are somehow negative because since COVID outbreak one of the possible sign to show that you are suspected is cough. Patient with cough tend to avoid the facility.” (CHC KI)*

### Health system challenges

There were several pre-existing health system challenges which continued to pose problems for service delivery during COVID, especially in relation to poor drug supplies at health facilities.

### Drug supplies and equipment

There was a poor supply of drugs for COVID and non-COVID patients as the health centres received drugs quarterly with frequent drug stock outs. It was challenging for non-COVID patients with chronic conditions visiting health facilities: drugs were not available when they were prescribed medicine and they found it difficult to go to the district town of Makeni to purchase those drugs. At this time, TB patients might not visit health services but relied rather on drug peddlers and self-medication. Common essential drugs were described as out of stock at CHCs, including for children and malaria, and emergency drugs at the hospital.

*“I think challenges for medication cut across, the medicines they supply are quarterly, mainly for free health. Since the last supply in November and up to date we do not have any supply. So there are challenges with essential drugs like amoxicillin, paracetamol.” (CHC KI)*

Routine vaccines were perceived as being in shorter supply since the advent of COVID, which was also accompanied by a change in the policy on allowing CHWs to administer rapid diagnostic testing for and drugs for malaria.

*“Like for the supplies for the marklate [vaccines] also, the Pentas and BCGs [vaccines] during the lockdown, even yesterday we went to Makeni for vaccines and they told us that there are no vaccines.” (CHC KI)*

Broader equipment and infrastructure challenges remained evident too, such as lack of commonly needed supplies and running water, alongside lack of housing at health centres for staff.

*“Even the instruments that we are using, we don’t have like the surgical instruments, as well as the materials- gauze, cotton wool, examination bed - and I have been calling for it to be changed to no avail. The one that is here is broken. Water is also a challenge; we don’t have good running water. For the toilet is manageable, but the housing is a challenge and majority of us are staying out of the CHC and even me as CHO I stay out of the CHC. The CHC is not fenced and not secured.” (CHC KI)*

### Supply of PPE

There was very poor availability of PPE (such as face masks) for health protection among health workers and patients at CHC level. Most of the available PPE (i.e., gloves and gowns) were left over from Ebola. Lacking PPE in the community caused health staff and CHWs to fear touching patients. Hospitals, however, had PPE but limited oxygen.

*“We don’t have face masks, PPE and we are in a crisis. It is like we are risking our lives and that of our families.” (CHC KI)*

### Health workforce challenges

Lack of staffing, staff not on payroll and high staff turnover was noted as a constraint by KIs, who also highlighted the lack of training on COVID prior to this intervention. As a result, at the beginning of the COVID outbreak, health workers were afraid to see patients, although this was reduced as protection and information was provided to them.

*“First when COVID just came, nurses and other health care workers were afraid of coming close to patients for fear of contracting the virus. Everyone was staying far away with wording like ‘please push a bit from me’ - a kind of social distancing. Sometimes, even if they call them, it was difficult for health care workers to come and attend to patients because they were not knowledgeable on how to handle suspected cases.” (CHC KI)*

### Facilitators

#### For adoption of guides

Given changeover of staff, an important element supporting impact of the desk guide and training was action taken by trainees to share resources and knowledge. For example, some CHOs shared the guide with the midwives, CHOs, and also SECHNs in their facility, which helped disseminate knowledge on prevention and treatment.

*“Even when I am not around in facility, the first thing that I do is to ensure that most of my staffs understand the guide. And they know how to use it. So anyone … in my office will see the guide on the table. And always we screen patient using the guide. We consult them using the guide and I think it has help us in identifying suspected cases.” (CHC KI)*

In the hospital, actions were also described to cascade knowledge to all groups of staff.

*“We also engage in popularization of these guidelines, starting from the gate, the security, porters, the cleaning staff and the doctors ourselves.” (Hospital KI)*

Ongoing training and encouragement to use the desk-guide were also mentioned as enablers.

*“When I continuing encouraging my staffs, I believe they will use the guide, and could be calling other cadres for training so that they too will have first-hand knowledge on COVID.” (CHC KI)*

#### For management of COVID

Key informants described active collaboration between the DHMT and the district COVID coordination body, to coordinate partners (including through daily briefings at the height of the crisis), share information on COVID and ensure active dissemination.

*“One of the reasons why I think we brought the improvement is the fact that joint venture of the hospital management and the DHMT through the district COVID coordination committee, and the social mobilization pillar has been very strong in disseminating information to the public to show that yes, COVID is not a death sentence. …” (Hospital KI)*

Partner investment supported refurbishing the treatment centre and testing for COVID.

*“We can do PCR now in the region I think, which is very good in managing of the patient…” (Hospital KI)*

There was a substantial focus on IPC measures, not just in facilities but also in markets and public places, likely building on memories and habits from the Ebola period.

*“Everybody is aware; you go to the market places you will see the hand washing bucket. You pass you will see everybody with facemask. We always emphasize it and with the help of the police. I think everybody is taking the preventive measures, which is good because prevention is better than cure.” (Hospital KI)*

Lots of community meetings were held to inform them about COVID and dispel fears.

*“Well the DHMT at primary care, they have held many community engagements. So we held them at the community centre and ask some of us to be there with the community people and explain to them that this COVID has come again and let us start follow the rules and regulations that govern the COVID.” (CHC KI)*

The community was sensitised that sick people could break the law enforced curfew and attend the hospital for care.

*“But with sanitization we do sensitize them, we talk to CHW, we talk to the town heads when we go for outreach, we tell them that Curfew is not for sick person, so who so ever is sick you have to take the person to the hospital so that he will get treatment and if they are referred earlier.” (Hospital KI)*

#### For maintenance of routine services

Routine clinical services, for example for non-communicable diseases, TB and malnutrition, continued to be offered as before, including referrals as required.

*“For malnutrition we still maintain the same criteria in doing the same thing, that is admitting children under 5 that are severely malnourished” (CHC KI)*

In some cases, services were reorganised in response to COVID –for example, there was change from weekly (immunization) services to everyday service to avoid overcrowding.

*“We are also doing our outreach activities. Immunization services are still going on and that social distancing is maintained and discouraged large crowd in the said activities. Before this time, they used to do that weekly (every Monday), but now every day when a pregnant woman or lactating mother comes.*” (CHC KI)

Outreach was also key to maintaining service use, as highlighted by multiple interviews. During outreach visits, health workers encouraged residents to visit health centres as needed. Health workers talked to them about COVID and the importance of hand washing and wearing of face masks. They conducted awareness raising campaigns to allay their fears, did radio discussions and other community engagement to reduce fears. Sensitization was reported to help improve awareness and service use by residents.

*“At first it was very disastrous, for two to three month we were not having patients, clients were afraid to come to hospital. . but with sanitization, mass sensitization, some have changed those thoughts. Journalists were coming to the hospital to interview us and they were playing our interview on air so the message reached the people. In those interviews we emphasize that the hospital is a safe place to come for treatment.” (Hospital KI)*

In particular, outreach campaigns improved the immunisation, ANC and under five care awareness and uptake, although adult curative services were reported to still be depressed.

*“The routine immunization now, people are accepting us at their villages when we went there for outreaches. So, before they ran away but now they accepted us when we went there for our routine outreaches and when someone defaulted, we will ask the mother to come with the child and she will come with the child, no hesitation.” (CHC KI)*

## Discussion

This study, although it was confined to one district of Sierra Leone, highlights the importance of providing adapted and context-specific guidelines for management of new emerging diseases like COVID, while continuing to emphasize enabling the delivery of existing services, whose absence will likely cause more loss of life than COVID itself [26, 27]. The desk guide and associated training were developed with national and district inputs and were seen as relevant and helpful by the participants, particularly in relation to increased health worker confidence in managing risks through IPC, educating their peers and patients and also detecting COVID and other conditions. However, we also highlight the major challenge of absence of testing facilities, which meant that few confirmed cases were recorded, and thus there was limited urgency given to COVID.

The legacy of Ebola, according to our findings, has been a mixed one in Sierra Leone: it appears to have resulted in rapid take-up of messages and practices relating to hygiene in facilities and public places. On the other hand, the trauma of Ebola replayed in people’s minds, with a strong unwillingness to enter places where they might be detected and detained. Both communities and health staff at least initially responded as if the disease were similar to Ebola, when in fact its spread and management were quite different (as is largely transmitted as aerosols rather than direct contact as with Ebola). In relation to practical external support, the epidemics were quite different too – as Ebola was focused in the region, districts perceived a significant increase in support which came via partners to the government. In the case of COVID, as the pandemic was global, international support was very limited in Sierra Leone.

As with other recent studies on COVID control [28, 29], and consistent with our recent research on NCD service delivery in Sierra Leone [24, 30], the continuing resource and health system challenges were also highlighted here – especially the poor drug supply, equipment and staffing situation, now augmented by lack of PPE – which acted as barriers to effectiveness of the desk-guides and indeed other measures to improve quality of care in Sierra Leone. Though the desk guides and tools were put online on the MoHS intranet, there was insufficient promotion of uptake elsewhere in Sierra Leone.

We do however note some positive adaptive strategies in the district, such as switching from weekly to daily immunisations, to avoid over-crowding. The expansion in outreach and public messaging also appears to have been effective as routine data suggests that utilisation – at least of preventive care, for which we have data – appears to have held up and anecdotal evidence for other (curative) services indicates a dip for few months but then a reasonably quick return to confidence and utilisation. This indicates encouraging resilience by both health system and communities [31, 32].

These findings are consistent with studies that have previously highlighted the need for more operational guidelines for health staff, especially at PHC level [24], the chronic stressors that the Sierra Leone health system faces [24] and the challenges relating to stigma and obstacles to isolation for communities in the context of infectious diseases in the region [22]. However, this is the first study that has specifically documented an attempt to improve management of COVID and ongoing services at primary care in a fragile state. It is important that the challenges arising are understood and addressed to prepare for further shocks and stressors to this and similar health systems.

### Study limitations

The study limitations include the small area covered – one district in Sierra Leone – as well as the relatively limited follow up period of three months after training. We also acknowledge the limited routine data which we were able to obtain from the HMIS, which focused on ANC and immunisation services but excluded curative care, such as outpatient visits and other services.

## Conclusion

This pilot study demonstrated the relevance and importance of case management guides and training modules, tailored to the specific context and its constraints, which were able to improve the confidence of health staff to manage their own and the community’s fears in the face of a new pandemic. In the case of Sierra Leone, previous epidemics, particularly Ebola, complicated this by both creating structures that could be revitalised but also assumptions and behaviours that were not adapted to the new disease (of COVID). Our study documents positive adaptations and resilience by health staff but also chronic system weaknesses (particularly for medicines, supplies and equipment) which must be urgently addressed before the next shock arrives.

### Supplementary Information

**Additional  file 1. **Key informant interview – topic guide for baseline, interim and follow up interviews.

**Additional file 2.** Checklist for facilities.

## Data Availability

The datasets generated and/or analysed during the current study are available from the corresponding author on reasonable request.

## Abbreviations

ANC: Antenatal Care
CHCs: Community Health Centres
CHOs: Community Health Officers
DHMT: District Health Management Team
DMO: District Medical Officer
HMIS: Health Management and Information System
IPC: Infection Prevention and Control
KI: Key Informant
MoHS: Ministry of Health and Sanitation
NCDs: Non-communicable Diseases
PHUs: Peripheral Public Health Units
PHC: Primary Health Care
RUHF: Health Research Global Health Research Unit on Health in Situations of fragility
WHO: World Health Organization

## Footnotes

1 Adapting from work done by COMDIS-HSD (http://comdis-hsd.leeds.ac.uk).

## References

[1] World Health Organisation. : Weekly epidemiological update on COVID-19–10 August 2022.https://www.who.int/publications/m/item/weekly-epidemiological-update-on-covid-19---10-august-2022. Accessed 10 Aug, 2022.

[2] World Health Organisation. : Sierra Leone Situation. https://covid19.who.int/region/afro/country/sl. Accessed on 12 Aug, 2022.

[3] Barrie MB, Lakoh S, Kelly JD, Kanu JS, Squire J, Koroma Z, Bah S, Sankoh O, Brima A, Ansumana R (2021). SARS-CoV-2 antibody prevalence in Sierra Leone, March 2021: a cross-sectional, nationally representative, age-stratified serosurvey. medRxiv.

[4] Grieco K, Yusuf Y. Rapid Country Study: Sierra Leone. Maintains. Oxford: Oxford Policy Management; 2020. https://www.opml.co.uk/files/Publications/A2241-maintains/final-2707-sierra-leone-covid-rapid-study-4-.pdf?noredirect=1.

[5] Diop BZ, Ngom M, Pougué Biyong C, Pougué Biyong JN (2020). The relatively young and rural population may limit the spread and severity of COVID-19 in Africa: a modelling study. BMJ Global Health.

[6] Hagan JE, Ahinkorah BO, Seidu AA, Ameyaw EK, Schack T (2020). Africa’s COVID-19 situation in focus and recent happenings: a mini review. Front Public Health.

[7] Njenga MK, Dawa J, Nanyingi M, Gachohi J, Ngere I, Letko M, Otieno CF, Gunn BM, Osoro E (2020). Why is there low morbidity and mortality of COVID-19 in Africa?. Am J Trop Med Hyg.

[8] Okonji EF, Okonji OC, Mukumbang FC, Van Wyk B (2021). Understanding varying COVID-19 mortality rates reported in Africa compared to Europe, Americas and Asia. Trop Med Int Health: TM IH.

[9] Post LA, Argaw ST, Jones C, Moss CB, Resnick D, Singh LN, Murphy RL, Achenbach CJ, White J, Issa TZ, Post LA, Argaw ST, Jones C, Moss CB, Resnick D, Singh LN, Murphy RL, Achenbach CJ, White J, Issa TZ, Boctor MJ, Oehmke JF (2020). A SARS-CoV-2 surveillance system in sub-Saharan Africa: modeling study for persistence and transmission to inform policy. J Med Internet Res.

[10] Villalonga-Morales A (2020). Why is Covid-19 epidemics no so intense in Africa?. Rev Esp Anestesiol Reanim.

[11] Zhao Z, Li X, Liu F, Zhu G, Ma C, Wang L (2020). Prediction of the COVID-19 spread in african countries and implications for prevention and control: a case study in South Africa, Egypt, Algeria, Nigeria, Senegal and Kenya. Sci Total Environ.

[12] Etteh CC, Adoga MP, Ogbaga CC (2020). COVID-19 response in Nigeria: Health system preparedness and lessons for future epidemics in Africa. Ethics Med Public Health.

[13] Bouchriti Y, Kabbachi B, Sine H, Naciri A, Kharbach A, Baba MA, Achbani A (2022). COVID-19 prevention and control interventions: what can we learn from the pandemic management experience in Morocco?. Int J Health Plann Manag.

[14] Clottey KNB, Debrah G, Asiedu L, Iddi S (2022). The short-term effect of the government of Ghana’s decision to open borders at the early-onset of the COVID-19 pandemic. Sci Afr.

[15] Akinbodewa AA, Odimayo MS, Ogundele OA, Ogunleye TO, Johnson OO, Lamidi OA, Akinmurele M, Oyebade OM (2021). Covid-19 pandemic: chronicle of responses and experiences of the infection prevention and control committee at a tertiary hospital in southwest Nigeria. Afr Health Sci.

[16] Ray S, Mash R (2021). Innovation in primary health care responses to COVID-19 in Sub-Saharan Africa. Prim Health care Res Dev.

[17] Dzinamarira T, Dzobo M, Chitungo I (2020). COVID-19: a perspective on Africa’s capacity and response. J Med Virol.

[18] Kanyangarara M, Allen S, Jiwani SS, Fuente D (2021). Access to water, sanitation and hygiene services in health facilities in sub-saharan Africa 2013–2018: results of health facility surveys and implications for COVID-19 transmission. BMC Health Serv Res.

[19] Anjorin AA, Abioye AI, Asowata OE, Soipe A, Kazeem MI, Adesanya IO, Raji MA, Adesanya M, Oke FA, Lawal FJ (2021). Comorbidities and the COVID-19 pandemic dynamics in Africa. Trop Med Int Health: TM IH.

[20] Nderitu D, Kamaara E (2020). Gambling with COVID-19 makes more sense: ethical and practical Challenges in COVID-19 responses in Communalistic Resource-Limited Africa. J Bioethical Inq.

[21] Haider N, Osman AY, Gadzekpo A, Akipede GO, Asogun D, Ansumana R, Lessells RJ, Khan P, Hamid MMA, Yeboah-Manu D (2020). Lockdown measures in response to COVID-19 in nine sub-saharan african countries. BMJ. Global Health.

[22] Schmidt T, Cloete A, Davids A, Makola L, Zondi N, Jantjies M (2020). Myths, misconceptions, othering and stigmatizing responses to Covid-19 in South Africa: a rapid qualitative assessment. PLoS ONE.

[23] Waya JLL, Ameh D, Mogga JLK, Wamala JF, Olu OO (2021). COVID-19 case management strategies: what are the options for Africa?. Infect Dis Poverty.

[24] Zou G, Witter S, Caperon L, Walley J, Cheedella K, Senesi RGB, Wurie HR (2020). Adapting and implementing training, guidelines and treatment cards to improve primary care-based hypertension and diabetes management in a fragile context: results of a feasibility study in Sierra Leone. BMC Public Health.

[25] Health Facility deskguide in the context of COVID-19 Sierra Leone. https://www.qmu.ac.uk/media/xhrf5rxf/deskguide-sl-hcs-160620.pdf. 2020.

[26] Kiarie H, Temmerman M, Nyamai M, Liku N, Thuo W, Oramisi V, Nyaga L, Karimi J, Wamalwa P, Gatheca G (2022). The COVID-19 pandemic and disruptions to essential health services in Kenya: a retrospective time-series analysis. Lancet Glob Health.

[27] World Health Organisation. : COVID-19 continues to disrupt essential health services in 90% of countries. https://www.who.int/news/item/23-04-2021-covid-19-continues-to-disrupt-essential-health-services-in-90-of-countries. Accessed on 1 May, 2022.

[28] Malik MA (2022). Fragility and challenges of health systems in pandemic: lessons from India’s second wave of coronavirus disease 2019 (COVID-19). Global Health Journal (Amsterdam Netherlands).

[29] Yusefi AR, Sharifi M, Nasabi NS, Rezabeigi Davarani E, Bastani P (2022). Health human resources challenges during COVID-19 pandemic; evidence of a qualitative study in a developing country. PLoS ONE.

[30] Witter S, Zou G, Diaconu K, Senesi RGB, Idriss A, Walley J, Wurie HR (2020). Opportunities and challenges for delivering non-communicable disease management and services in fragile and post-conflict settings: perceptions of policy-makers and health providers in Sierra Leone. Confl Health.

[31] Blanchet K, Diaconu K, Witter S, Bozorgmehr K, Roberts B, Razum O, Biddle L (2020). Understanding the resilience of health systems. Health Policy and Systems Responses to Forced.

[32] Haldane V, De Foo C, Abdalla SM, Jung A-S, Tan M, Wu S, Chua A, Verma M, Shrestha P, Singh S, Haldane V, De Foo C, Abdalla SM, Jung A-S, Tan M, Wu S, Chua A, Verma M, Shrestha P, Singh S, Perez T, Tan SM, Bartos M, Mabuchi S, Bonk M, McNab C, Werner GK, Panjabi R, Nordström A, Legido-Quigley H (2021). Health systems resilience in managing the COVID-19 pandemic: lessons from 28 countries. Nat Med.

