# Supplementary material for: Learning from implementation of a COVID case management desk guide and training: a pilot study in Sierra Leone
Source: BMC Health Serv Res. 2023 Sep 25;23:1026. doi: 10.1186/s12913-023-10024-6 (PMC10518973; doi:10.1186/s12913-023-10024-6)
Supplement: Supplementary file 1 — Additional file 1. Key informant interview – topic guide for baseline, interim and follow up interviews. [file 12913_2023_10024_MOESM1_ESM.docx]

**Supplementary file 1**

**Key informant interview – topic guide for baseline, interim and follow up interviews**

**DHMT- eg:** District Medical Officer and communicable disease control officer, Social Mobilisation, Community Engagement Officer or District Health Sister

Tracking epidemiological and health systems responses

1. How was the COVID situation last month?
2. What is your role in the COVID response and how long have you been in it?
3. How has COVID impacted on routine services, such as routine prevention programs or maternal and child health programs in the district? Has there been change over the last 1 month?
4. Have you been able to actualise government policies on COVID-19?
   1. If so what? What has not been possible and why?
   2. What are your recommendations to improve preparedness and responsiveness for epidemics in the district?
5. How have you been engaged with the command centres in the district?
   1. Has the collaboration been good? How/why?
   2. What could be done to improve it?

Tracking the use of desk-guides and their impact on routine health services

1. Are you aware of the desk-guide developed by our team (COMAHS and Queen Margaret University)? If no, what other material has been used with respect to COVID-19 and what has been most useful and why? If yes, continue to answer the following questions:
   1. Have the module and desk-guides helped the CHOs in the CHCs? If so how?
   2. Was there any other training or material from other organisations or DHMT that supported you and if so how?
   3. Was there anything in the desk guide that you think needed changing or adapting?
   4. What changes have been made since/if they are used?
   5. Has there been any changes to the way the CHCs are triaging patients with respect to COVID?
   6. Do you think there has been a change to the clinical care of patients with suspected COVID at the CHC’s since introducing the guide?
   7. Has there been any change to the care of routine patients without suspected Covid due to the guide for e.g. hypertension, malaria, ANC?
   8. Have you noticed changes in how the community perceives the CHC and its preparedness to address COVID, since the guide has been introduced?
   9. Barriers and enablers to the actions in the desk guide (if the desk-guide is not well implemented, why?)

**CHC staff –CHOs and SECHNs**

Tracking epidemiological and primary care responses

1. About your role
2. What is the COVID situation in the last month?
3. What has changed in the last month with respect the way primary care is responding to the COVID outbreak (enablers of and barriers)?

- Public health measures implemented in the CHCs
- Isolation and Treatment of suspected cases
- Routine prevention programs e.g. child immunisation, ANC
- Routine clinical services e.g. TB, malnutrition services, non-communicable diseases?
- Any challenges with PPE or IPC materials?
- Any challenges with medication for COVID and non COVID patients?
- How have national policies affected your area of work, what did you do in responses; why/why not?

1. What is going well, and what not so well?

Tracking the use of desk-guides and their impact on routine health services

1. Have you read the desk guide and module? If not, why?
2. If you have read and used the deskguide, can you share with us the following:
   1. How relevant are they? strengths/weaknesses?
   2. What did you learn?
   3. Any elements which need changing?
   4. Who did you share them with?
   5. How have they been used (based on the content of desk-guide)?

- Identification and diagnosis of COVID cases
- Management of suspected cases and close contacts, if any
- Referral of the patients to the district hospital
- Education of home isolated cases, if any
- Communication with the district health workers and CHWs
- Protection of yourself at work?
- Facility management（e.g., screening and isolation area management)
- Management and reallocation of healthcare staff
- Infection Prevention and Control (IPC), including disinfection etc: why/why not providers may implement these and how it all relates to perceptions of risk (contextual factors like limited availability of space, culture of care related ones – e.g. mask wearing compromising patient communication)
  1. What changes have been made since/if they are used (in the last month)?
  2. Barriers and enablers to the actions in the desk guide (if the deskguide is not well implemented, why?)
  3. How is the routine healthcare for hypertension/diabetes (NCD) care continuing/ affected by COVID? How can the desk guide help with challenges?
  4. How is the routine healthcare for ANC and child health been affected by COVID? How can the desk guide help with challenges?

1. Did you receive any further support, supervision or follow up visits to help you in the COVID response? How was this helpful if at all?

**District hospital staff : as possible of medical superintendent, the matron, doctor, OPD CHO or nurse**

Tracking epidemiological and hospital responses

1. What is your role in the COVID response and how long have you been in it?
2. How has COVID impacted on routine services, such as routine prevention programs or maternal and child health programs in the district? Has there been change over the last 1 month?
3. Have you been able to actualise government policies on COVID-19? If so what? What has not been possible?
4. Do you have challenges with IPC? Please describe them
5. Do you have challenges with PPE? Please describe them
6. Do you have challenges with provision of treatment to COVID and non COVID patients? E.g. Ventilators, medication? Please describe them

Tracking the use of desk-guides and their impact on routine health services (mostly relevant to the Medical superintendent or doctor)

1. Have you read the Management Guidelines for District Hospitals During COVID-19? If no, ask why or if there was any other material or training, they used that was supportive and how did it bring about any change; if yes, continue to answer the following questions:
2. How relevant are they?
3. What did you learn, if anything?
4. Any elements which need adding?
5. Who did you share them with?
6. How have they been used?

- Screening, identification and diagnosis of COVID cases
- Report of the suspected and confirmed cases
- Treatment of suspected cases and manage close contacts
- Protection of yourself
- Facility management（e.g., screening and isolation area management)
- Drugs and supplies including laboratory test materials and PPEs
- Management and reallocation of healthcare staff
- Hospital infection prevention and control (IPC)

1. What changes have been made since/if they are used (in the last month)?
2. Barriers and enablers to the actions in the Hospital management in COVID guide

(if the deskguide is not well implemented, why?)

1. What has been the effect of the guides on the routine healthcare, for non COVID related illness such as TB and NCD care by COVID?
2. Was there any other material or training that was useful and how was it supportive and how did it bring about change?
