# Supplementary material for: Learning from implementation of a COVID case management desk guide and training: a pilot study in Sierra Leone
Source: BMC Health Serv Res. 2023 Sep 25;23:1026. doi: 10.1186/s12913-023-10024-6 (PMC10518973; doi:10.1186/s12913-023-10024-6)
Supplement: Supplementary file 2 — Additional file 2. Checklist for facilities. [file 12913_2023_10024_MOESM2_ESM.docx]

**Additional file 2. Checklist for facilities**

**Background**

The activity started on the 21^st^ July 2020 and ended on the 31^st^ July 2020. The two weeks activity was carried out with the 22 CHC in Bombali where the five CHO’s are mentoring. The activity was jointly carried out by the CHO’s and the RUHF social Mobilization officer in Bombali District.

The checklist for the field visit was designed to look at the challenges Health facility workers are faced within the 22 facilities. The checklist is divided in three broader components/sections such as:

1. Section A. Basic Information
2. Section B. COVID information
3. Section C. NCD information.

The purpose of the field was to do an assessment on the situation within our intervention facilities that will guide us in designing our training materials for an effective COVID and NCD work.

| **No** | **Facilities within District** | **Facilities within Karena** |
| --- | --- | --- |
|  | Police Clinic | Batkanu |
|  | Masongbo | Kamalo |
|  | Makeni | Kamakwie |
|  | Rokonta | Kamawonie |
|  | Mapaki | Kathanta Yimbor |
|  | Panlap |  |
|  | Bikolo |  |
|  | Kagbaneh |  |
|  | Kalangba |  |
|  | Mataboi |  |
|  | Gbendembu |  |
|  | Kamabai |  |
|  | Makeni |  |
|  | Rokula |  |
|  | Kagbere |  |
|  | Kamaranka |  |
|  | Mabunduka |  |

**SECTION B: COVID19**

| **Assessment** | | **Response** |
| --- | --- | --- |
| 1. Please record in the last month | 1. Number of patient visits in the last month |  |
|  | 1. Number of COVID-19 suspected cases in the last month? |  |
|  | 1. Number of confirmed cases in the last month? |  |
|  | 1. Number of patients with fever? |  |
|  | 1. Number of patients with loss of smell or loss of taste? |  |
|  | 1. Number of patients with cough? |  |
| 1. If possible observe the triage process? | 1. What is working well? |  |
|  | 1. What challenges are there? |  |
| 1. If possible observe the separation into COVID and non-COVID areas: | 1. What is working well? |  |
|  | 1. What challenges are there? |  |
| 1. Observe the Infection Prevention Control: | 1. What is working well? |  |
|  | 1. What challenges are there? |  |

| 1. Comment on the isolation unit | 1. Use |  |
| --- | --- | --- |
|  | 1. Preparation for COVID patients |  |
| 1. How has covid made it more challenging to manage patients with non covid related problems eg ANC, immunization, chronic disease management?   **To be asked on first visit** | |  |
| 1. What may be the solutions? | |  |

**SECTION C: NCD.**

Ensure all staff of all cadres have been trained how to use the desk guide and treatment card on your first visit

| PHU staff trained? |  |
| --- | --- |
| Are treatment cards available |  |
| Number of treatment cards in use |  |
| Is desktop guide being used? |  |
| Blood pressure machine available? |  |
| Blood pressure machine functional |  |
| Are there medications available to treat Hypertension |  |
| Glucometer available? |  |
| Glucometer functional? |  |
| Glucometer strips available? |  |
| Medication available to treat Diabetes |  |
| Number of referrals from other PHUs |  |
| Main challenges in treating patients with NCDs |  |
